# Biofluid Metabolomics and Lipidomics of Mice Exposed to External Very High-Dose Rate Radiation

**DOI:** 10.3390/metabo12060520

**Published:** 2022-06-04

**Authors:** Evan L. Pannkuk, Evagelia C. Laiakis, Guy Garty, Shivani Bansal, Brian Ponnaiya, Xuefeng Wu, Shanaz A. Ghandhi, Sally A. Amundson, David J. Brenner, Albert J. Fornace

**Affiliations:** 1 Department of Oncology, Lombardi Comprehensive Cancer Center, Georgetown University Medical Center, Washington, DC 20057, USA; ecl28@georgetown.edu (E.C.L.); sm3451@georgetown.edu (S.B.); af294@georgetown.edu (A.J.F.J.); 2 Department of Biochemistry and Molecular & Cellular Biology, Georgetown University Medical Center, Washington, DC 20057, USA; 3 Center for Metabolomic Studies, Georgetown University, Washington, DC 20057, USA; 4 Radiological Research Accelerator Facility, Columbia University, Irvington, NY 10032, USA; gyg2101@cumc.columbia.edu; 5 Center for Radiological Research, Columbia University Irving Medical Center, New York, NY 10032, USA; bp156@cumc.columbia.edu (B.P.); xw2696@cumc.columbia.edu (X.W.); sg2423@cumc.columbia.edu (S.A.G.); saa2108@cumc.columbia.edu (S.A.A.); djb3@cumc.columbia.edu (D.J.B.)

**Keywords:** biodosimetry, lipidomics, metabolomics, ionizing radiation, very high-dose rate, mass spectrometry

## Abstract

High-throughput biodosimetry methods to determine exposure to ionizing radiation (IR) that can also be easily scaled to multiple testing sites in emergency situations are needed in the event of malicious attacks or nuclear accidents that may involve a substantial number of civilians. In the event of an improvised nuclear device (IND), a complex IR exposure will have a very high-dose rate (VHDR) component from an initial blast. We have previously addressed low-dose rate (LDR, ≤1 Gy/day) exposures from internal emitters on biofluid small molecule signatures, but further research on the VHDR component of the initial blast is required. Here, we exposed 8- to 10-week-old male C57BL/6 mice to an acute dose of 3 Gy using a reference dose rate of 0.7 Gy/min or a VHDR of 7 Gy/s, collected urine and serum at 1 and 7 d, then compared the metabolite signatures using either untargeted (urine) or targeted (serum) approaches with liquid chromatography mass spectrometry platforms. A Random Forest classification approach showed strikingly similar changes in urinary signatures at 1 d post-irradiation with VHDR samples grouping closer to control samples at 7 d. Identical metabolite panels (carnitine, trigonelline, xanthurenic acid, N6,N6,N6-trimethyllysine, spermine, and hexosamine-valine-isoleucine-OH) could differentiate IR exposed individuals with high sensitivity and specificity (area under the receiver operating characteristic (AUROC) curves 0.89–1.00) irrespective of dose rate at both days. For serum, the top 25 significant lipids affected by IR exposure showed slightly higher perturbations at 0.7 Gy/min vs. 7 Gy/s; however, identical panels showed excellent sensitivity and specificity at 1 d (three hexosylceramides (16:0), (18:0), (24:0), sphingomyelin [26:1], lysophosphatidylethanolamine [22:1]). Mice could not be differentiated from control samples at 7 d for a 3 Gy exposure based on serum lipid signatures. As with LDR exposures, we found that identical biofluid small molecule signatures can identify IR exposed individuals irrespective of dose rate, which shows promise for more universal applications of metabolomics for biodosimetry.

## 1. Introduction

A set of coordinated terrorist attacks in the United States on 11 September 2001 led to the first renewed interest in establishing funding for ionizing radiation (IR) medical countermeasures and rapid testing for IR exposures since the time of the Cold War [1]. In addition to potential malicious attacks with improvised nuclear devices (INDs) or dirty bombs, we have since witnessed a nuclear accident at the Fukushima Daiichi Nuclear Power Plant in 2011 and attacks on Ukrainian nuclear power plants during a Russian invasion in 2022 that further highlight the need for high-throughput biodosimetry following IR exposures. Adding to the expansion of biodosimetry tools has been the advancement of several -omics approaches developed in the life sciences, including transcriptomics, proteomics, and metabolomics/lipidomics [2]. Metabolomics involves the collective analysis of small molecules (typically polar compounds < 1 kDa) while the similar lipidomics refers to analysis of the non-polar water insoluble fraction (typically compounds < 1.5 kDa) [3]. Both strategies lead to a plethora of data useful for determining biological damage from IR exposures; for example, changes in polar tricarboxylic acid cycle intermediates in urine are routinely perturbed post-irradiation while the bis-allylic structure of polyunsaturated lipids in blood make them excellent markers for the extent of damage from reactive oxygen species (ROS) introduced from indirect IR effects [4]. Ideally, the top candidates from both of these disciplines can be combined into a single rapid multiplex assay useful in dose reconstruction and be effective irrespective of the complex nature associated with radiation exposures (e.g., dose rate), combined injury, genetic predisposition, etc. A particular recent focus of our group has been elucidating the effects of dose-rate on biofluid signatures and its potential to alter predictive model power [5,6,7,8,9].

Questions concerning the effects of very high-dose rates of VHDR (here 7 Gy/s) vs. conventional dose rates (~1 Gy/min) are applicable in the realm of homeland security [10]. In the potential detonation of a 10 kt nuclear device, there will be three primary types of radiation fields of concern. The initial blast will consist of a mix of neutron and gamma radiation delivered at VHDR, with less effects at longer distances. Exposure to the VHDR irradiation from this initial blast may be accompanied by subsequent exposure from delayed radiation, which will consist of an external exposure to groundshine and fallout and be delivered at a much lower dose rate (<1 Gy/day) that can last for days. Third, internal emitter radiation, of primary concern the radioisotope ^137^Cs, can continue for weeks if chelating agents are not used [11]. As our previous research addressed effects of internal emitters or nuclear fallout on dose reconstruction [5,7,8,9,12], further research is also needed in the VHDR range.

In this study, we examined how VHDR rate exposures would compare to a reference dose rate and may affect the utility of biofluid small metabolite panels that may be used in biodosimetry. Male 8–10-week-old C57BL/6 mice were exposed to a cumulative dose of 3 Gy using a reference dose rate of 0.7 Gy/min or a VHDR of 7 Gy/s. We identified changes in the urinary metabolome using an untargeted metabolomics approach and the serum lipidome using a targeted approach on liquid chromatography (LC) mass spectrometry (MS) platforms. We found a similar response between dose rates in urine, where higher perturbations were observed at 1 d, but the VHDR group response returned back to background at 7 d. Identical metabolite panels were able to differentiate mice on both days with excellent to good specificity and sensitivity based on the area under the receiver operating characteristic (AUROC) curves irrespective of dose rate (AUROC ≥ 0.9 excellent, ≥0.8 good). A critical component of these panels included a novel compound, Hex-V-I, a metabolite that was previously described by our group [8]. For serum, most lipids showed a similar response between groups with slightly higher perturbation observed in the reference dose rate group compared to VHDR. As with urine, identical lipid panels showed excellent sensitivity and specificity at 1 d; however, the 7 d samples could not be differentiated from control samples for a 3 Gy exposure with either dose rate.

## 2. Results and Discussion

### 2.1. Untargeted Metabolomics: Urine

**Figure 1:**
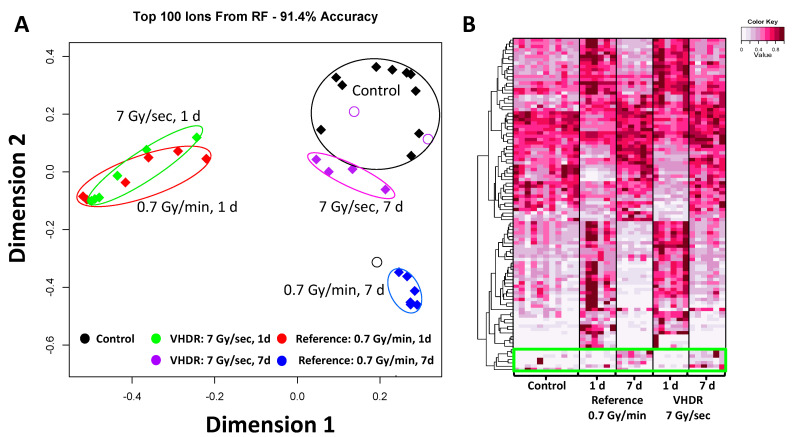
Results from the Random Forests analysis in urine. (**A**) A multidimensional scaling (MDS) plot of the top ranked 100 ions in ESI+ (classification accuracy 91.4%). The highest perturbation for both dose rates occurred at 1 day (d). Mice exposed to very high-dose rate (VHDR) ionizing radiation (IR) (7 Gy/s) returned closer to control levels at 7 d compared to mice exposed to the reference dose rate (0.7 Gy/min). (**B**) A heatmap of the top ranked 100 ions in ESI+ showed that the divergence of the 1 d samples includes both metabolites occurred at higher and lower concentration compared to the control in urine. Each metabolite is scaled by the maximum intensity value of that metabolite in the data set. While most metabolite levels returned to control levels at 7 d, a small subset showed increased levels (green box).

The resultant data matrix consisted of 8933 spectral features in ESI^+^ mode and 6796 spectral features in ESI^−^ mode. Visualization of the top ranked 100 ions (classification accuracy of 91.4%) from the ESI^+^ dataset showed similar groupings of the 1 d samples irrespective of dose rate compared to the control (Figure 1). The 7 d small molecule signatures were more similar to the control compared to 1 d, with higher perturbation observed at the reference dose rate (0.7 Gy/min) compared to the VHDR (7 Gy/s) cohort. Although urinary signatures typically show increased separation from non-irradiated individuals during the initial few days (~3 days) and begin to return to basal levels within a week [13], it is important to recognize dose effects are still high enough at 7 d time points to be practical for biodosimetry [14].

**Figure 2:**
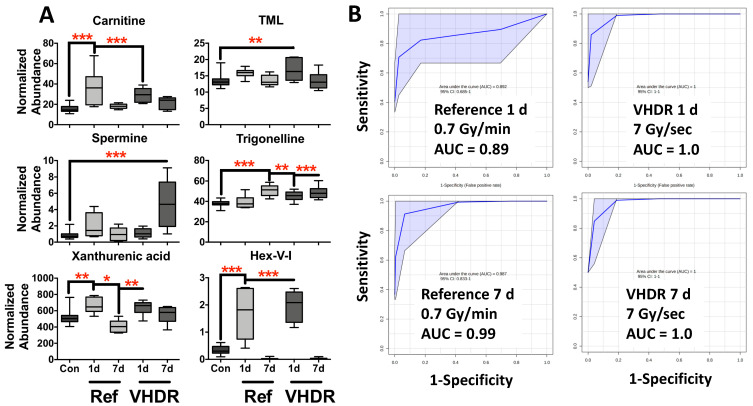
(**A**) Metabolite levels in mouse urine after exposure to the reference dose rate (0.7 Gy/min) or very high-dose rate (VHDR) (7 Gy/s) at 1 and 7 days (d). These metabolites have been identified in previous radiation experiments and were used to determine the area under the receiver operating characteristic curves (AUROC). (**B**) ROC curves at 1 d (N6,N6,N6-trimethyllysine (TML), carnitine, Hex-V-I) at 7 d (trigonelline, carnitine, Hex-V-I, spermine) show excellent to good sensitivity and specificity irrespective of dose rate. (* *p* < 0.05, ** *p* < 0.01, *** *p* < 0.001 determined by a one-way ANOVA and a Dunnett’s multiple comparison test with *p* values corrected for multiple comparisons of post-irradiation samples to the control, Means ± S.E.; AUROC classification: excellent ≥ 0.9, good ≥ 0.8).

Increases were observed at 1 d in compounds typically identified post-irradiation, including carnitine (*p* < 0.001, reference FC = 2.4, VHDR FC = 1.9), TML (*p* = 0.006, VHDR FC = 1.3), and xanthurenic acid (*p* < 0.001, reference FC = 1.3, VHDR FC = 1.3) (Figure 2, Table 1). All three of these metabolites have been identified in a previous study analyzing urine from mice exposed to low-dose rate IR (≤1 Gy/day) and reference dose rate exposures [8], as well as several other studies examining post-irradiation effects on urinary signatures [15,16]. TML, and carnitine in particular, are among the most consistently altered metabolites post-irradiation and indicate perturbation to fatty acid β oxidation. Interestingly, xanthurenic acid, a product of tryptophan catabolism, may show differential effects from dose rate. Identification in LDR studies typically show reduced urinary concentrations [6,8,17,18], however, here we observed an increase at 1 d. A slight decrease occurred here at 7 d (reference FC = 0.8) highlighting our previous conclusions that involvement of the host microbiota and diet in producing tryptophan metabolites will complicate their use in biodosimetry panels. Increased levels of trigonelline were only observed in the VHDR 1 d cohort (*p* < 0.001, VHDR FC = 1.2), but for both doses at 7 d (*p* < 0.001, reference FC = 1.4, VHDR FC = 1.3). Interestingly, levels of spermine showed higher fold increase at 7 d in the VHDR group (*p* < 0.001, VHDR FC = 6.3), which was not observed in the reference dose rate group. Several reports have implicated dysregulated metabolic conversion of spermidine to spermine or *N*^1^-acetylspermidine (see [19]), and it may be partially attenuated by deficient p53 activity [20] or age [21]. Perturbation to polyamine levels due to IR exposure is interesting, given the role of this pathway in DNA oxidation and apoptosis.

We previously identified a novel metabolite (Hex-V-I) in urine from mice exposed to both reference dose rate and LDR exposures [8], where up to 80-fold increases were observed at 1–3 d after the reference dose rate. Here, we show high fold increases after VHDR exposure (*p* < 0.001, VHDR FC = 5.7) in addition to the reference dose rate (*p* < 0.001, reference FC = 5.0) providing further evidence of the utility of Hex-V-I as a radiation marker at earlier time points and that it is animal facility independent (Figure 2). At 7 d, levels of Hex-V-I were depleted to a level below detection. While the origin of Hex-V-I is still under investigation, its inclusion into multiplex assays increases the sensitivity and specificity of biodosimetry models. We and others demonstrated the differences that the dose rate may have on bio-physiological response [6,22]. However, in terms of biodosimetry there is a need to determine more universal biomarkers to simplify assays. As with LDR exposures, we observed that metabolites involved in energy metabolism and the newly described Hex-V-I that are present in urine provide excellent candidates for dose reconstruction (Figure 2). Identical metabolite panels provided excellent to good sensitivity and specificity for both the reference dose rate and VHDR exposure at 1 d (Hex-V-I, carnitine, and TML) (reference AUROC = 0.89, VHDR AUROC = 1.0) and excellent at 7 d (Hex-V-I, carnitine, trigonelline, and spermine) (reference AUROC = 0.99, VHDR AUROC = 1.0). Furthermore, these panels failed to classify post-irradiated groups based on dose rate, providing additional evidence that urinary metabolite panels can be utilized independent of dose rate for biodosimetry (Figure S2). 

### 2.2. Targeted Lipidomics: Serum

Several untargeted lipidomic studies have investigated irradiation effects in several models including cell cultures [23], murines [12,24,25,26], NHPs [27,28,29], and humans [30] while the initial targeted lipidomic studies have primarily been limited to commercially available kits or eicosanoid profiling [31,32,33,34,35]. More recently, targeted lipid studies utilizing multiplex MRM assays showed that combinations from 416 lipids in blood could be used for modelling IR exposure between 4–72 h for 0–8 Gy in rats with excellent sensitivity and specificity [36]. As we typically see an increased number of lipid compounds changes post-irradiation in serum vs. polar compounds, we chose to utilize a targeted lipidomics approach in the current study. Here, we show that although changes in serum lipid levels may be dose rate dependent, the overall changes are similar enough for excellent classification at earlier time points (1 d) after a 3 Gy exposure, but later time points (7 d) may require higher doses for adequate separation.

Some of the more pronounced effects from radiation exposure in the serum are found as dynamic responses in lipid compounds. We found several glycerolipids (TAGs and DAGs) that were significantly lower at 1 d in both groups but were back to control levels at 7 d. While hydrolytic cleavage of these compounds can produce FFAs that will play further roles in inflammation, here the FFAs showed similar trends to the glycerolipids. Trends in glycerolipids observed in the current study may be due to a possible dietary component, e.g., not eating due to handling stress, so they were removed from further analysis. Of the remaining 427 lipids measured, 14 broad classes (2 CEs, 10 HexCers, 9 SMs, 5 acyl-carnitines, 5 LPAs, 2 LPCs, 12 LPEs, 1 LPI, 8 PCs, 9 PEs, 7 ePEs, 4 PGs, 16 PIs, and 2 PSs) had at least one lipid species that was significantly different from the control in at least one experimental group (Supplementary File S1).

**Figure 3:**
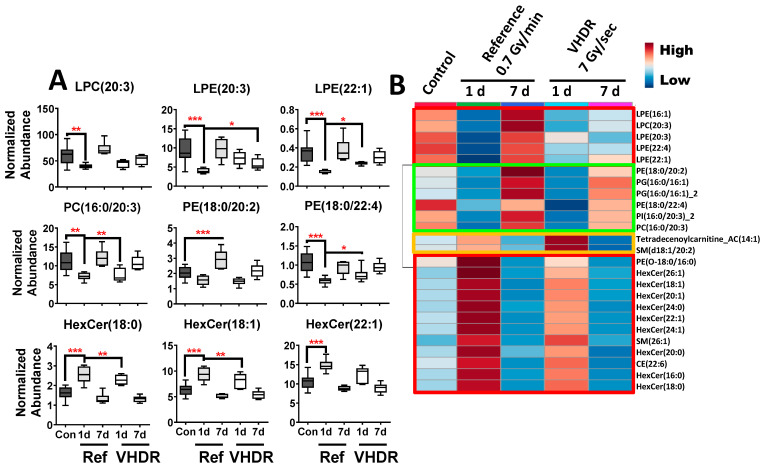
(**A**) Lipid concentrations in mouse serum after exposure to the reference dose rate (0.7 Gy/min) or very high-dose rate (VHDR) (7 Gy/s) at 1 and 7 days (d) identified from the heatmap in panel B. (**B**) A heatmap of the top 25 lipids shows that several lysoglycerophospholipids, glycerophospholipids, and hexosylceramides (red boxes) display similar patterns between dose rates with slightly higher fold changes in the reference dose rate cohort. (* *p* < 0.05, ** *p* < 0.01, *** *p* < 0.001 determined by a one-way ANOVA and a Dunnett’s multiple comparison test with *p* values corrected for multiple comparisons of post-irradiation samples to the control, Means ± S.E.).

**Figure 4:**
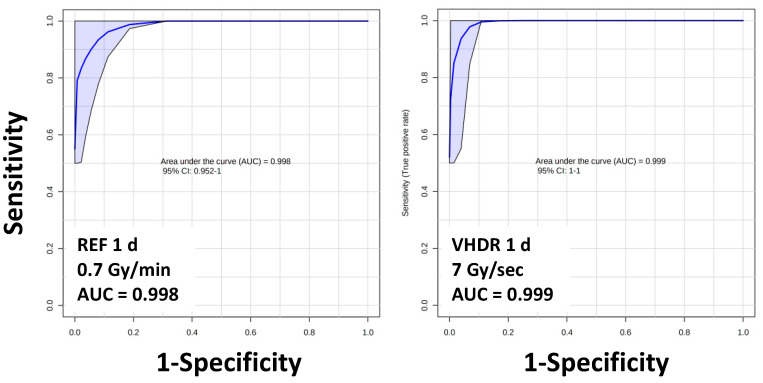
Area under the receiver operating characteristic curves (AUROC) values for serum lipids show excellent sensitivity and specificity (AUROC > 0.9) irrespective of dose rate. Lipid panel includes HexCer(16:0), HexCer(18:0), HexCer(24:0), SM(26:1), and LPE(22:1).

A heatmap of the top 25 lipids was composed of 5 lysoglycerophospholipids (LGP) (LPE and LPC), 6 glycerophospholipids (GPs) (PC, PE, PI, and PG), 9 HexCers, CE(22:6), 2 SMs, tetradecenoylcarnitine, and ePE(O-18:0/16:0) (Figure 3). Although the lipids were found statistically significant after exposures of both dose rates, primarily at 1 d, higher fold changes were observed for LGPs, HexCers, an CE in the reference dose rate compared to the VHDR group at 1 d (red boxes) with levels returning closer to the control group by 7 d. Relatively similar changes were observed in GPs irrespective of dose rate (green box) and slightly higher perturbations were observed in tetradecenoylcarnitine and SM(d18:1/20:2) after the VHDR exposure (yellow box). Although slightly different fold changes were observed between the experimental groups, an identical lipid panel (HexCer16:0], HexCer[18:0], HexCer[24:0], SM[26:1], LPE[22:1]) was able to achieve excellent sensitivity and specificity irrespective of dose rate (reference AUROC = 0.998, VHDR AUROC = 0.999) at 1 d (Figure 4). Serum lipid panels could not differentiate the 7 d group from the control group for either dose rate after a 3 Gy exposure, which we have observed in past studies at lower doses (2 Gy in NHPs), but increased fold changes occur at higher doses (10 Gy in NHPs) and may be more useful for identifying individuals exposed to critically high IR levels [27].

One of the lesser characterized lipid classes in terms of biodosimetry is the HexCers. Hexosylceramides are sphingolipids that consist of a ceramide backbone with a neutral sugar headgroup (i.e., glucose or galactose). Formation of HexCer starts with a ceramide precursor that is converted to a glucosylceramide primarily in the Golgi with transfer of a glucose from UDP-glucose by glucosylceramide synthase (GCS, EC: 2.4.1.80) [37]. Due to the importance of ceramides in cellular apoptosis, increased GCS expression increases ceramide glycosylation to make cells resistant to apoptosis. Disruption of GCS expression has been observed to lead to p53 driven apoptosis in p53 mutant cells [38]. Decreased plasma levels of HexCer were observed in humans undergoing pelvic radiation therapy [39]. We found several HexCer species that showed higher fold increases at 1 d after reference dose rates compared to VHDR, then returned to basal levels at 7 d. Another lipid subgroup which has received less attention are CEs, which are conjugates of cholesterol with long chain fatty acids that are important in lipid storage and transport rather than serving as a structural membrane component. Polyunsaturated (PUFA) CEs, typically containing 18:2, 20:4, or 22:6 acyl chains, can also be enzymatically or non-enzymatically oxidized and can form more complex peroxides [40]. Previous experiments have reported perturbation to CE(20:4) and CE(22:6) levels at higher doses in NHPs (10 Gy) and rats (8 Gy) [27,36] with a temporal change in NHPs showing lower concentrations at earlier time points (4 h), then increasing at later time points (7–10 d) [28]. These changes may be partially dose rate dependent, as in this study we observed increases in CE(22:6) at 1 d following the reference dose rate exposure and a decrease in CE(20:4) following the VHDR exposure (Supplementary File S1). As previous lipidomic studies investigating products from lipid oxidation following radiation exposure have focused on FFAs and GPs, studies on CE oxidation may also reveal interesting biomarkers [24,30,31].

Also critical for the AUROC sensitivity and specificity in the current study were LPE(22:1) and SM(26:1). Responses of LGPs after IR exposure have been reported in detail after reference dose rates, where increases in lipid species potentially involved in inflammation (LPC[20:4], LPC[22:6], LPE[20:4], LPE[22:6]) increase in a dose dependent manner [27,36,41] but will exhibit a temporal dynamic response [28]. Similarly, several SM species have been identified post-irradiation using reference dose rates and can also display a temporal and dose dynamic response. SMs are also derived from ceramides similar to HexCers; however, they are formed through the combination of CDP-choline and N-acylsphingosine by ceramide cholinephosphotransferase (EC: 2.7.8.3) or a ceramide and phosphatidylcholine by sphingomyelin synthase (EC: 2.7.8.27). Radiation damage can also target the SM pool by activating sphingomyelinases that increase ceramide production and initiate apoptosis [42].

## 3. Material and Methods

### 3.1. Animal Models and Radiation Exposure

These experiments were approved by the Columbia University Institutional Animal Care and Use Committee (IACUC; approved protocol AABA5458) and were conducted under all relevant federal and state guidelines. Male 8 to 10-week-old C57BL/6 mice were obtained from Charles River Laboratories (Frederick, MD, USA) and were irradiated using the FLASH irradiator at the Radiological Research Accelerator Facility [43] (Figure S1). This novel irradiator is based on a Clinac 2100C (Varian Medical Systems, Corona, CA, USA) where the pulse delivery is controlled using in-house software. All irradiations were performed using 9 MeV electrons with no scatter or flattening filter. For these experiments, mice were placed in a 72 mm × 41 mm × 41 mm acrylic box in which air holes had been drilled (The Container Store, Coppell, TX, USA). For 0.7 Gy/min irradiations, mice (*n* = 6) were individually placed at 120 cm above the Clinac head and irradiation delivered at 3.25 pulses per second. In this configuration, 3 Gy was delivered in 580 pulses. For 7 Gy/s mice (*n* = 6) were individually placed 20 cm above the Clinac head (Figure S1) and dose delivered at 180 pulses/s after allowing 20 s where the acceleration and electron source were both on but operated asynchronously so that no beam is delivered. In this configuration, 3 Gy was delivered in 78 pulses. Dosimetry was performed prior to irradiation using a NIST-traceable Advanced Marcus Ion Chamber (AMIC) and Unidos E electrometer (PTW, Freiburg, Germany). Verification of dosimetry was performed using OBT3 radio-chromic film (Ashland Specialty Chemicals, Wayne, NJ, USA).

At 1 or 7 d post-irradiation, spot urine (>100 µL) was collected from control and irradiated mice prior to euthanasia. Urine samples were immediately stored at −80 °C and transported to GU on dry ice. Mice were subsequently euthanized by CO_2_ inhalation and blood was collected by cardiac puncture. Serum samples were prepared using BD Microtainer Tube (REF 365967) with ~100 µL of whole blood added to each tube, kept at room temperature for 30 min, then spun at 1300× *g* at 4 °C for 10 min. Serum was stored at −80 °C and shipped on dry ice to Georgetown University Medical Center.

### 3.2. Chemicals

We used Fisher Optima^TM^ grade reagents for all sample preparation and LC mobile phases (Fisher Scientific, Hanover Park, IL, USA). Chemical standards for urine metabolomics included debrisoquine sulfate and 4-nitrobenzoic acid for internal standards, and carnitine, trigonelline hydrochloride, xanthurenic acid, Nε,Nε,Nε-trimethyllysine hydrochloride, and spermine were obtained from Sigma-Aldrich (St. Louis, MO, USA). Hexosamine-valine-isoleucine-OH (Hex-V-I) was synthesized by Expert Synthesis Solutions (London, ON, Canada) and structure confirmation has been previously published [8]. Chemical standards for serum lipidomics included EquiSPLASH^®^ LIPIDOMIX, (*d*_7_) 15:0/18:1 phosphatidic acid, (*d*_7_) 18:1 cholesteryl ester (CE) (Avanti Polar Lipids Inc., Alabaster, AL, USA), the free fatty acid (FFA), dihydroceramide (DCER), hexosylceramide (HexCer), and lactosylceramides (LCER) internal standard kits for the Lipidyzer™ Platform (Sciex, Framingham, MA, USA), and the NIST plasma Standard Reference Material (SRM) 1950.

### 3.3. Untargeted Metabolite Profiling in Urine

Samples were prepared and analyzed as previously described [14,44]. Briefly, urine (20 μL) was deproteinized (80 μL 50% cold acetonitrile) with internal standards (2 μM debrisoquine [M+H]^+^ = 176.1188; 30 μM 4-nitrobenzoic acid [M-H]^−^ = 166.0141), vortexed, incubated on ice (10 min), and centrifuged for 10 min (max speed, 4 °C). A 1 μL aliquot of each sample was combined before processing for a quality control (QC) sample and then prepared as above. Samples were injected (2 μL) into a Waters Acquity Ultra Performance Liquid Chromatography (UPLC) with a BEH C18 1.7 μm, 2.1 × 50 mm column and coupled to a Xevo^®^ G2-S quadrupole time-of-flight (QTOF) MS (Waters, Milford, MA, USA). Positive and negative electrospray ionization (ESI) data-independent modes were used for data acquisition with leucine enkephalin ([M+H]^+^ = 556.2771, [M-H]^−^ = 554.2615) as Lock-Spray^®^. Operating conditions for ESI were: capillary voltage 2.75 kV, cone voltage 30 V, desolvation temperature 500 °C, desolvation gas flow 1000 L/Hr. Mobile phases consisted of the following: solvent A (water/0.1% formic acid [FA]) and solvent B (acetonitrile/0.1% FA). The gradient for urine was (solvent A and B) 4.0 min 5% B, 4.0 min 20% B, 5.1 min 95% B, and 1.9 min 5% B at a flow rate of 0.5 mL/min, column temp 40 °C. Blanks and QC samples were run after every 10 samples.

### 3.4. Targeted Lipid Profiling in Serum

We used a targeted lipid profiling assay designed to analyze several lipid groups including: cholesteryl esters (CE), cholesterol, ceramides (Cer), hexosylceramides (HexCer), lactosylceramides (LCER), dihydroceramides (DCER), sphingomyelins (SM), acylcarnitines, diacylglycerides (DAG), triacylglycerides (TAG), monoacylglycerides (MAG), free fatty acids (FFA), phosphatidic acids (PA), lysophosphatidic acids (LPA), phosphatidylcholines (PC), ether-linked phosphatidylcholines (ePC), lysophosphatidylcholines (LPC), phosphatidylinositols (PI), lysophosphatidylinositols (LPI), phosphatidylethanolamines (PE), ether-linked phosphatidylethanolamines (ePE), lysophosphatidylethanolamines (LPE), phosphatidylglycerols (PG), and phosphatidylserine (PS). A 20 μL aliquot of serum was mixed with 5 v. (100 μL) of extraction buffer (isopropanol) containing internal standards (see chemicals) per the manufacturer’s instructions. Samples were vortexed for 30 s, incubated on ice for 30 min, and then centrifuged for 10 min (max speed, 4 °C). The supernatant was transferred to MS vial for LC-MS analysis. Quality control samples of both a pooled sample and the NIST SRM 1950 plasma mix were run every 10 samples. The NIST SRM 1950 plasma was prepared as above. Samples were kept at 15 °C on a SIL-30 AC auto sampler (15 °C) until analysis (Shimazdu, Columbia, MD, USA).

We injected samples (5 µL) onto a Xbridge amide column (3.5 µm, 4.6 × 100 mm) (Waters, Milford, MA, USA) maintained at 35 °C before analysis with a Sciex QTRAP 5500 Mass Spectrometer (Sciex, Framingham, MA, USA). Lipids were detected by multiple reaction monitoring (MRM) transitions in both positive and negative ionization mode. The MRM transitions for statistically significant lipids are listed in Supplementary File S1. Mobile phases consisted of the following: solvent C (95% acetonitrile/5% water with 10 mM ammonium acetate) and solvent D (50% acetonitrile/50% water with 10 mM ammonium acetate). The gradient for serum was (solvent C and D) initial gradient 100% C, 3.0 min 99.9% C, 3.0 min 94% C, 4.0 min 25% C, 6.0 min 0% C, 6.0 min equilibrate back to 100% C, at a flow rate of 0.7 mL/min. Source and gas setting were as follows: temperature =  550  °C, nebulizing gas  =  50 and heater gas  =  60, curtain gas  =  30, CAD gas  =  medium, ion spray voltage  =  5.5 kV in positive mode and −4.5 kV in negative mode.

### 3.5. Data Processing, Statistical Analysis, and Marker Validation

For urine, we manually inspected raw data files in MassLynx v.4.1 (Waters Corporation, Milford, MA, USA) and used Progenesis QI (Nonlinear Dynamics, Newcastle, UK) for pre-processing, including peak alignment and picking. Normalization was performed using a software chosen QC chromatogram as an alignment reference and normalizing to the “normalize to all compounds function”. Initial identifications for spectral features were determined to ±10 ppm error of the monoisotopic mass using the human metabolome database (HMDB) [45] and the METLIN MS/MS empirical library [46]. We first screened for statistically significant spectral features using two-sample comparisons for features present at ≥70% in both groups (Welch’s *t*-test) or <70% presence in a single group (Barnard’s test) using a false discovery rate corrected (Benjamini–Hochberg step-up correction procedure) *p* value of <0.10 as previously described with the software MetaboLyzer [47]. Pre-urine samples were also compared as above to remove statistically significant features not due to IR exposure. All spectral features of interest were validated to a metabolomics standards initiative (MSI) level 1, i.e., we matched the accurate *m/z*, retention time, and tandem MS (5–50 V ramping collision energy) fragmentation patterns to pure standards [48,49]. Validated compounds in urine were plotted in GraphPad Prism 9.2.0 to check for outliers (ROUT Q = 1%) and equal variances (Bartlett’s test) (GraphPad Software, La Jolla, CA, USA). As there were no statistically different differences between pre-exposure samples they were grouped and compared to the irradiated samples with a one-way ANOVA with a post-hoc Dunnett’s multiple comparison test with *p* values corrected for multiple comparisons. Multidimensional scaling (MDS) plots and heatmaps were generated using the machine-learning algorithm Random Forests programmed in R v.2.15.2 [50], as this is a prominent method within the -omics field for handling high dimensional datasets. The AUROC values were determined by generating ROC curves in MetaboAnalyst 5.0, with a Random Forests classification method for combined metabolites [51,52].

For the targeted serum lipid assay, the qlm file was imported into MultiQuant v.2.0 (Sciex, Framingham, MA, USA) and peak areas were visually inspected before exporting to Excel. Lipids present in the pooled QC sample with a coefficient of variation >25% were removed and not further considered. The remaining lipids were normalized to their respective internal standard. We screened the resultant data matrix in SAS 9.4 (SAS, Cary, NC, USA) using the proc glm function and a post-hoc Dunnett’s test. Lipids of interest were plotted in GraphPad Prism 9.2.0 to check for outliers (ROUT Q = 1%) and equal variances (Bartlett’s test) (GraphPad Software, La Jolla, CA, USA). Controls consisted of sham-irradiated mice and compared to the irradiated samples with a one-way ANOVA with a post-hoc Dunnett’s multiple comparison test with *p* values corrected for multiple comparisons. The AUROC values were determined by generating ROC curves in MetaboAnalyst 5.0, with a Random Forest classification method for combined metabolites, along with heatmaps with ANOVA of the top 25 lipid compounds [51,52].

## 4. Conclusions

The ultimate goal of these studies is to develop a simple analytical method that can easily be adapted across clinical labs so that multiple testing centers can be quickly established in an emergency situation and process approximately half a million samples per day [53]. The complex nature of an IR exposure from an IND will inherently consist of different dose rates beyond the conventional 1 Gy/min exposures that are typically employed in laboratory settings. Different dose rates may have both dependent vs. independent effects, therefore it is paramount to determine if dependent effects alter dose reconstruction accuracy for biodosimetry. Here, we found similar results to our previous studies on differential effects from variable dose rate and genotypic differences, where the effects of IR exposure are so consequential that exposed individuals are easily identified from non-exposed individuals. These results are encouraging, as high variation in metabolite signatures due to dose rate or genotypic differences could complicate its utility in biodosimetry. In terms of dose rate, fold changes in urinary metabolites following IR exposure are typically great enough that identical metabolites can be used for classification irrespective of a delivery rate spanning seconds to days. Having identical signatures could simplify data interpretation in emergency medical situations. Serum provides a rich source of structurally diverse lipids that are sufficiently IR sensitive to provide excellent classification, albeit this structural diversity may lead to rather complicated data interpretation [36]. Overall, the results from these studies are encouraging and biofluid small molecule signatures for biodosimetry will remain a viable option following a radiological emergency.

## Figures and Tables

**Table 1:** Validated urinary metabolites that significantly changed in mice exposed to reference dose rates (0.7 Gy/min) and very high-dose rates (7 Gy/s).

Metabolite	Adduct	RT	Experimental (*m*/*z*)	Calculated (*m*/*z*)	Mass Error	HMDB	Formula	MS/MS Fragments		
					(ppm)			Fragment 1	Fragment 2	Fragment 3
Spermine	H+	0.22	203.2234	203.2236	0.8	0001256	C_10_H_26_N_4_	129.1377	112.1148	84.0854
TML	H+	0.27	189.1605	189.1603	1.1	0001325	C_9_H_20_N_2_O_2_	130.0874	84.0812	60.0791
Hex-V-I	H+	1.31	393.2247	393.2234	3.3	162421477 *	C_17_H_32_N_2_O_8_	309.1785	216.1211	150.0859
Carnitine	H+	0.29	162.1128	162.1130	1.2	0000062	C_7_H_16_NO_3_	103.0402	85.0286	60.0815
Xanthurenic acid	H+	0.89	206.0453	204.0297	0.2	0000881	C_10_H_7_NO_4_	178.0499	160.0394	132.0447
Trigonelline	H+	0.29	138.0556	138.0555	0.7	0000875	C_7_H_7_NO_2_	110.0610	94.0646	92.0509

* Pubchem CID.

## Data Availability

These metabolomics data have been submitted to Metabolomics Workbench with project identifiers ST002176 and ST002175.

## Supplementary Materials

The following are available online at https://www.mdpi.com/article/10.3390/metabo12060520/s1, Supplemental File S1: Lipids that were significantly different from the control group in mice exposed to a reference dose (0.7 Gy/min) or a very high-dose rate (VHDR) (7 Gy/s) at a total of 3 Gy at 1 and 7 days. Highlighted cells correspond to groups significantly different from the control group. Figure S1: (A) Study design and sample size. (B) Photo of mouse being irradiated in the FLASH irradiator. Figure S2: Area under the receiver operating characteristic curves (AUROC) values for urine show poor sensitivity and specificity (AUROC < 0.6) when comparing post-irradiated groups from VHDR and reference dose rate. This provides further evidence that select urine markers will change independently of dose rate. ROC curves contain identical metabolites at 1 d (N6,N6,N6-trimethyllysine [TML], carnitine, Hex-V-I) and at 7 d (trigonelline, carnitine, Hex-V-I, spermine).
