# Supplementary material for: Biofluid Metabolomics and Lipidomics of Mice Exposed to External Very High-Dose Rate Radiation
Source: Metabolites. 2022 Jun 4;12(6):520. doi: 10.3390/metabo12060520 (PMC9228171; doi:10.3390/metabo12060520)
Supplement: Supplementary file 1 [file metabolites-12-00520-s001.zip › Supplementary_Figures.pdf]

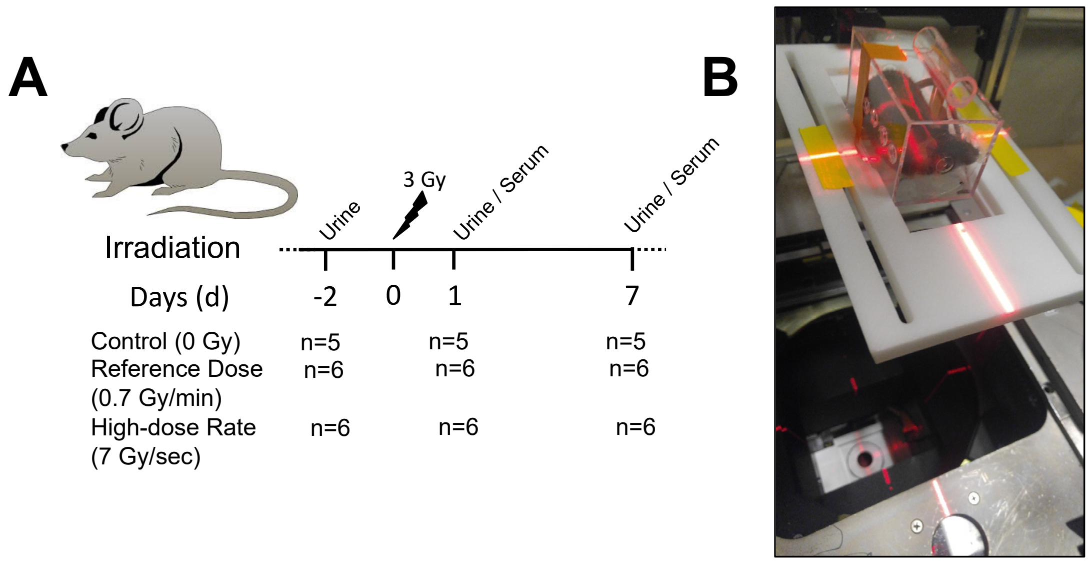

Supplementary Figure S1. (A) Study design and sample size. (B) Photo of mouse being irradiated in the FLASH irradiator.

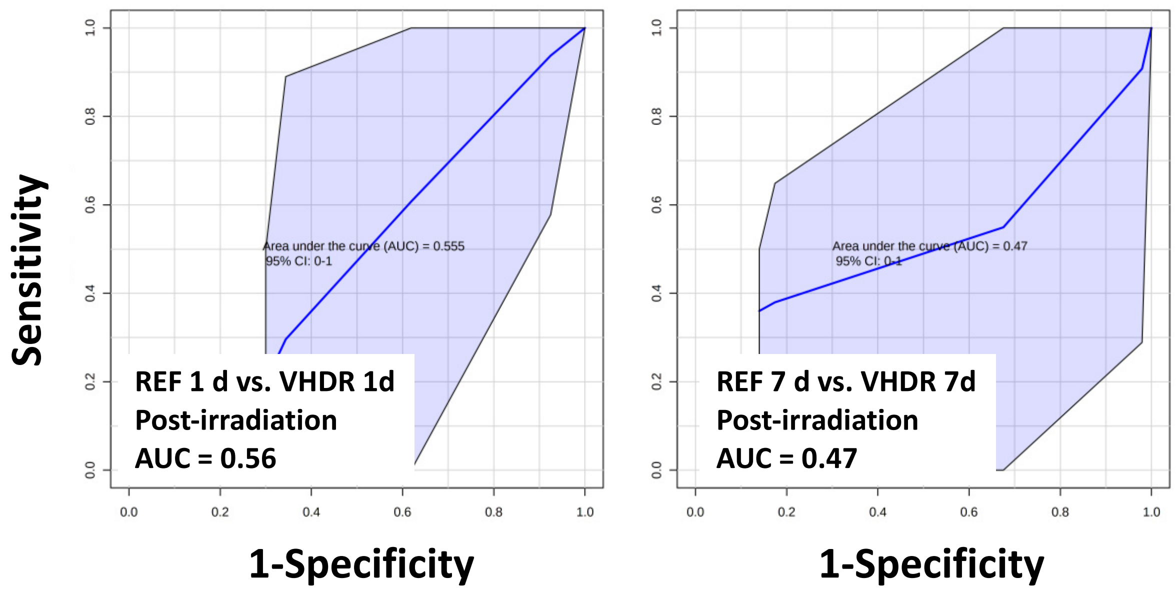

Supplementary Figure S2. Area under the receiver operating characteristic curves (AUROC) values for urine show poor sensitivity and specificity (AUROC < 0.6) when comparing post-irradiated groups from VHDR and reference dose rate. This provides further evidence that select urine markers will change independently of dose rate. ROC curves contain identical metabolites at 1 d (N6,N6,N6-trimethyllysine [TML], carnitine, Hex-V-I) and at 7 d (trigonelline, carnitine, Hex-V-I, spermine). These metabolomics data have been submitted to Metabolomics Workbench with project identifiers ST002176 and ST002175.
